# Gender Inequality in Low- and Middle-Income Countries: Associations with Parental Physical Abuse and Moderation by Child Gender

**DOI:** 10.3390/ijerph191911928

**Published:** 2022-09-21

**Authors:** Julie Ma, Andrew C. Grogan-Kaylor, Shawna J. Lee, Kaitlin P. Ward, Garrett T. Pace

**Affiliations:** 1 Department of Social Work, University of Michigan-Flint, 303 E. Kearsley St., Flint, MI 48502, USA; 2 School of Social Work, University of Michigan, 1080 South University Avenue, Ann Arbor, MI 48109, USA

**Keywords:** child maltreatment, child physical abuse, family violence, UNICEF Multiple Indicator Cluster Surveys

## Abstract

Gender inequality perpetuates women’s economic insecurity and a culture of violence. Parental distress caused by economic pressure may increase violence against children. High levels of gender inequality and interpersonal violence may contribute to higher levels of physical abuse. Using an ecological perspective, this study examines the association of country-level gender inequality and household-level parental physical abuse, and the moderating role of child gender in this association in low- and middle-income countries. We used data on over 420,000 households from the UNICEF Multiple Indicator Cluster Surveys and country-level indicators from the United Nations Development Program Human Development data. We employed multilevel logistic regression to examine the association between gender inequality with the log-odds of physical abuse after accounting for country- and individual-level covariates. In order to more fully explore our results, we calculated predicted probabilities of abuse for several scenarios. The results indicated that higher levels of gender inequality were associated with higher probabilities of physical abuse. This association was stronger for female children than for male children. The probabilities of abuse by child gender were indistinguishable, although rates of physical abuse converged as gender inequality increased, at a statistically marginal level. These findings indicate that macro-level interventions that reduce gender inequality are necessary to prevent and reduce child physical abuse.

## 1. Introduction

Gender inequality is a major challenge for global public health that undermines women’s rights and empowerment. The Gender Inequality Index (GII) is a composite measure of country-level gender inequity, which the United Nations Development Programme (UNDP) introduced in 2010 [1]. The GII encompasses three dimensions of women’s status in society: reproductive health, access to higher education and political empowerment, and labor market participation. In countries with higher levels of GII, women are subject to discriminatory gender roles that restrict their access to educational and employment opportunities and result in a lack of financial independence. Gender inequality is a social determinant of harmful norms that legitimize gender-based violence [2,3]. As the burden of violence rests disproportionately on vulnerable populations, violent norms and behaviors against women may propagate violence targeting children in the family context [4]. A cross-national study has uncovered the associations between gender inequality and country-level rates of child maltreatment [5]. What is missing from prior research is whether the GII is associated with rates of child physical abuse at the household level, and the moderating role of child gender in these associations. We utilized the UNICEF Multiple Indicator Cluster Surveys (MICS) to assess these associations. 

### 1.1. Gender Inequality in LMICs

The roots of gender inequality are multiple, occurring as a result of a complex interplay of factors [6]. The GII used in the current study is an aggregated indicator that captures a number of these dynamics, including women’s health (via maternal mortality rate and adolescent birth rate); empowerment (via education outcomes, such as percentage of females with secondary education and female shares of parliamentary seats); and labor market participation (via female labor force participation rates). Overall, research documents that women in LMICs have lower levels of health, empowerment, and labor market participation, both in comparison to men within their country and in comparison to men and women in more economically advantaged contexts [6]. Gender gaps in outcomes tend to be the highest in low-income countries with the poorest socio-economic conditions [7]. Many LMICs have made significant strides in remedying gender inequalities [8]. Even so, women in the most disadvantaged LMICs consistently face considerable barriers related to gender equality and low access to resources, stymying life outcomes for themselves and their children. 

In the domain of health, the GII captures adolescent birth rate and maternal mortality as unique factors that contribute to gender inequality. There is overwhelming evidence to suggest that women and children in LMICs experience a much higher burden of adolescent pregnancy and maternal death than their counterparts in higher income countries [9,10]. An estimated 12 million adolescents in LMICs give birth each year [11], accounting for 95% of the adolescent births globally [12]. Adolescent pregnancy is a major risk factor for maternal mortality [11]. Indeed, the global burden of maternal mortality disproportionately falls on women in LMICs. The WHO reports that LMICs account for a staggering 94% of all maternal deaths due to childbirth [13]. A large population-based study of women in LMICs found that lower levels of education were associated with greater risk for maternal mortality, pointing to the complex interplay of factors that are measured in the GII [9]. 

In the domain of empowerment, the GII measures female and male education attainment, with gender gaps in educational attainment showing a “male bias,” or an inequality in which men attain higher rates of education than women. In general, both female and male children in countries with the lowest gross national income are much less likely to attend preschool and to have lower quality stimulation in their home environments [14]. This inequality is further exacerbated by gender. Research has demonstrated the association between country-level gross domestic product (GDP) and gender inequality with a strong male bias in education at the lowest levels of GDP [6]. That is, as GDP increases, rates of male and female college enrollment tend to equalize and in fact disappear at the highest levels of GDP [6]. In reference to political power across the globe, although the legislative representation of women has increased, women are still underrepresented in government. One study showed that women currently hold 26% of the legislative seats around the world [15]. Women’s empowerment through political representation is much lower in LMICs, and lower levels of political representation have been linked to adverse maternal outcomes, including higher levels of neonatal, infant, and under-5 mortality in LMICs [16,17].

In the domain of labor market participation, the GII measures female and male labor force participation. Women in LMICs suffer from higher levels of economic hardship and gender inequality than women in economically advanced countries. Furthermore, nearly 1 in 4 girls across the globe are neither employed nor in education and training, compared to 1 in 10 boys [18]. Overall, research suggests heterogeneity in labor market participation of women in LMICs, with factors such as household economic conditions and the supply of more educated women factoring into women’s opportunities for labor market participation [7].

To summarize, global data on gender inequality and human development indicate that girls and women in LMICs face high levels of gender inequality. The negative impact of these persistent and pervasive forms of gender inequality is tremendous, given that the majority of the world’s population (75%) reside in low- and middle-income countries.

Gender inequality restricts women’s economic, social, and educational opportunities relative to men and creates a subordinate social status for women. Research shows that gender discrimination contributes to gender-based norms that legitimize violence against women and that such norms are associated with family violence [2,3]. A robust line of research has revealed the cross-level association between macro-level gender inequality and women’s individual risk of male-based violence in the family context, including intimate partner violence [19,20]. Estimates have found high rates of gender-based violence in LMICs, where the GII is reported to be higher than in higher income countries, such that 26% of women aged 15 years or older in LMICs report exposure to husband-to-wife violence [21]. Furthermore, rape and sexual violence against women are common across the globe. Approximately 1 in 20 female adolescents between ages 15 and 19 have experienced forced sex; rates of child marriage and female genital mutilation are high [18,21,22].

### 1.2. Parental Physical Abuse in LMICs

Structural gender inequality increases the risk of parental physical abuse [5]. Parent-to-child physical abuse is the most pervasive form of violence in the lives of children. The World Health Organization (WHO) defines physical abuse as acts “which result in actual or potential physical harm from an interaction or lack of an interaction, which is reasonably within the control of a parent or person in a position of responsibility, power or trust” [23]. Worldwide, parental engagement in severe physical abuse—which includes beating a child up or hitting a child on the head, ears, or face—is at an alarming rate of 17% [24,25]. The prevalence of severe physical abuse reaches over 40% in several countries [24,25]. The high level of parental violence that children in LMICs are exposed to is a risk for poor social-emotional development [26,27] and exacerbates global inequalities in children’s development. 

### 1.3. Gender Inequality and Parental Physical Abuse

Given that most caregivers of children are women, the United Nations Children’s Fund (UNICEF) recognizes the significant role of women’s empowerment and social status in shaping the health and wellbeing of children [28]. The WHO suggests that gender inequality may be a macro-level predictor of child maltreatment [4]. Two potential mechanisms may explain the link between gender inequality and parental violence against children. The family stress model proposes that economic hardship acts as a severe stressor in the family context [29]. Unequal access to resources, power, and opportunities caused by structural gender discrimination weakens women’s economic security and life opportunities. This limits women’s caregiving capacity and may increase the use of harsh and abusive parenting practices [30,31,32]. 

A second mechanism is explained by the cultural spillover hypothesis. Gender inequality is associated with violence against women [2,3,33]. Through violence socialization processes, attitudes that endorse gender-based violence may spill over to norms that legitimize violence against children [34,35]. Notably, two cross-national studies of developing and transitional countries demonstrated bivariate associations between country-level gender inequality and country-level rates of child physical abuse [5,36]. 

However, there are limitations to existing research that has examined the link between gender inequality and physical abuse. Although the socio-ecological framework emphasizes the interactions between micro- and macro-level factors in shaping human behavior, extant studies have only focused on aggregated, country-level rates of parental physical abuse. Such use of aggregated data may be subject to an “ecological fallacy” in which country-level associations are substantially different, or even reversed, from individual-level associations [37,38]. Thus, appropriate multi-level models with individual-level data are warranted [38]. Moreover, these studies have not controlled for potential parent- and family-level confounders, such as caregiver attitudes toward physical punishment and family socio-economic factors, which prior research has identified as predictors of parental physical abuse [39]. Thus, the association between gender inequality and physical abuse of children in LMICs remains relatively unexplored, especially using multilevel data that contains information at both the country- and family-level.

### 1.4. Possible Differences by Child Gender

Gender inequality negatively affects both male and female children, however patterns may differ for male and female children. For example, in LMICs, girls face a higher risk of discrimination than boys in health care and educational settings and are subject to alarmingly high rates of violence that stem from gender inequality [40]. In contrast, other evidence suggests that boys may be subject to more parental physical abuse than girls in LMICs [24,39,41,42], although one meta-analysis of the global prevalence of parental physical abuse did not find significant gender differences [43]. With somewhat conflicting evidence regarding possible differences in parenting processes by child gender, no research to date has examined whether the associations between gender inequality and parental violence may differ for female and male children in LMICs. On one hand, the associations of gender inequality and exposure to parental violence may be more pronounced for girls who are likely to be more strongly and directly affected by discriminatory norms and practices than boys [44]. It is also possible that the associations of gender inequality with parental violence are not dependent on child gender.

### 1.5. The Present Study

The purpose of the current study was to uncover the associations of country-level gender inequality with caregiver-reported child physical abuse. We examined the differential influence of country-level, household-level, and individual caregiver- and child-level factors on children’s exposure to physical abuse. Furthermore, we examined the potential moderating role of child gender in the associations between gender inequality and parental physical abuse. Our first hypothesis was that when gender inequality increases that the probability of physical abuse would be higher. Our second hypothesis was that gender inequality would have a greater effect on rates of physical abuse for female children than for male children.

## 2. Methods

### 2.1. Data and Sample

Country-level information on gender inequality came from the United Nation Development Programme (UNDP) Human Development Report Office. UNDP releases progress in key dimensions of human development annually for 191 UN member states, such as the Gender Inequality Index (GII). 

We used household-level data on parenting behaviors from Round 4 and Round 5 of the Multiple Indicator Cluster Surveys (MICS) that were developed and administered by UNICEF. MICS surveys used a multistage sample design to select household samples that reflect the population that they draw from, representing counties and provinces of their country. The first stage of sampling involved selecting census enumeration areas, which were generally selected from a master sampling frame or a country’s recent census. In the second stage, households were selected from the enumeration areas using random systematic sampling and formed the survey clusters. Each survey cluster included 20 to 25 households. Household interviews were conducted in-person with the head-of-households, which was a man in most households, their spouse or a knowledgeable adult who was age 18 or older. Household respondents provided data on household socio-economic characteristics and physically abusive behaviors. MICS Round 4 and Round 5 randomly selected one child in the household aged 2 to 14 (Round 4) or aged 1 to 14 (Round 5) to be the reference child for the survey section on parenting behaviors. Trained interviewers administered the in-person surveys after obtaining oral consent. Data for the present study were derived from every publicly released, nationally representative survey in MICS Round 4 and Round 5 as of August 2020 that had data on parenting behaviors and the GII available. The analysis sample included 424,414 households in 51 countries. 

### 2.2. Measures

Parental Physical Abuse. Caregiver physical abuse was measured by two dichotomous items (1 = “yes”; 0 = “no”) in the Household Survey using a UNICEF-modified version of the Parent-Child Conflict Tactics Scale (CTS). The CTS is widely recognized for its reliability, validity, and appropriateness for child maltreatment research with international and cross-cultural populations [45]. Household respondents were asked whether any adult in the household used the following behaviors to the child in the preceding month of the interview: (1) “Beat [child] up, that is, hit [child] over and over as hard as one could” and (2) “Hit or slapped [child] on the face, head, or ears.” We combined these two items to create a binary variable that indicated whether physical abuse “occurred” (=1) or “did not occur” (=0) in the past month. Operationalization of physical abuse in this study was consistent with the CTS by Straus et al. [45] and a prior MICS study [39] that identified the items measuring two physically aggressive parenting behaviors in this study as severe physical maltreatment. 

Gender Inequality. Country-level gender inequality was measured by the Gender Inequality Index (GII), a widely accepted global gender index calculated by the UNDP [1]. The GII is a composite measure that aggregates gender disparities in three aspects of human development—reproductive health (i.e., maternal mortality ratio and adolescent birth rate), empowerment (i.e., proportion of parliamentary seats occupied by females and proportion of adult females and males with at least some secondary education), and labor market participation (i.e., labor force participation rates of female and male populations)—in each country. The GII ranges from 0 to 1 with higher GII values indicating more disparities between females and males in the country. To aid interpretation of results, our analyses used a rescaled GII that ranges from 0 to 100. 

Covariates. In our analyses, we controlled for country, household, and parent characteristics that have been identified as correlates to parental physical abuse in prior studies [41]. Gross Domestic Product (GDP) of each country was retrieved from the World Bank [46] and rescaled to range from 0 to 100. Household respondents provided responses to the following questions in the household survey in MICS: whether the respondent believed physical punishment is necessary to raise children properly (0 = “no”; 1 = “yes”; 2 = “don’t know/no opinion”), number of household members (capped at 50 members), respondent’s relationship to child (0 = “biological parent”; 1 = “grandparent”; 2 = “other”), whether the household respondent is male, and education level of the household respondent (0 = “none”; 1 = “primary”; 2 = “secondary or more”). Child characteristics included child gender (0 = “male”; 1 = “female”) and child age in years. We also controlled for the household wealth index, which was a cumulative measure of household’s assets and living standards that places household’s relative wealth into quintiles, area of residence (0 = “rural”; 1 = “urban”), and MICS round (0 = “Round 4”; 1 = “Round 5”).

### 2.3. Analytic Approach

First, we estimated descriptive statistics using sampling weights. Subsequently, we estimated four multilevel logistic regression models, controlling for country-level clustering, to examine the associations between gender inequality and caregiver physical abuse. Model 1 estimated the odds of physical abuse as a function of country-level GII and MICS round. Model 2 added household characteristics to Model 2. In Model 3, we estimated the odds of physical abuse as a function of GII and child gender, after controlling for caregiver, child, and household characteristics. Model 4 estimated the moderation between GII and child gender. After estimating the multilevel logistic regression analyses with the GII and child gender interaction, we calculated predicted probabilities of abuse to more fully explore these associations. Analyses were performed using Stata SE version 15 [47]. 

## 3. Results

**Figure 1:**
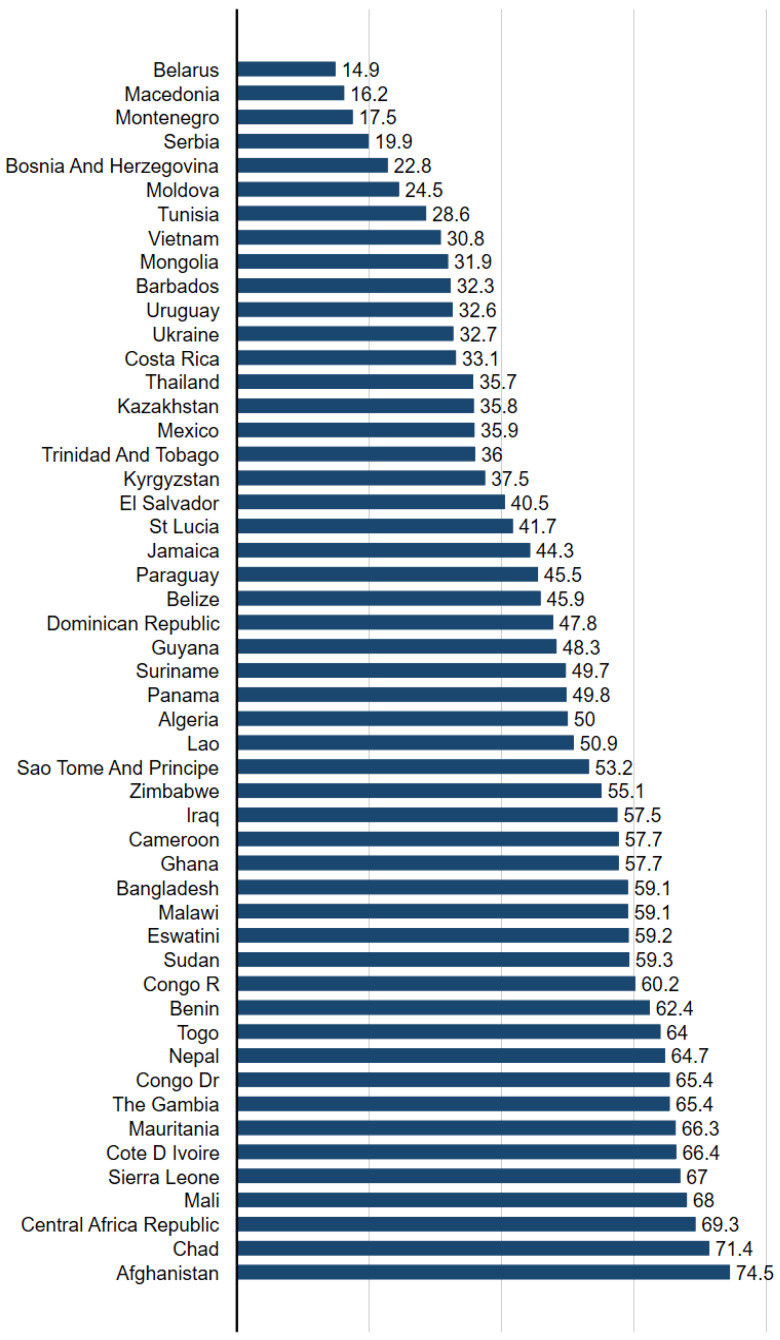
Gender Inequality Index Across 51 Countries in Analysis Sample.

Table 1 reports weighted descriptive statistics for all variables. Overall, 7.7% of children were exposed to physical abuse in the past month. As shown in Figure 1, across the 51 countries, GII ranged from 14.9 to 74.5, with an average of 52.3. 

Results from multilevel logistic regression are reported in Table 2. Model 1 indicated that higher levels of GII are associated with higher odds of caregiver physical abuse (OR = 1.051; *p* < 0.001) after adjusting for country-level GDP and MICS round. Model 2 showed a positive association between GII and caregiver physical abuse (OR = 1.048; *p* < 0.001) after controlling for household characteristics (i.e., household wealth quintile, urban residence, and number of household members). Model 3 was the full model that provides estimates of country-, household-, and individual parent- and child-level characteristics. In this model, higher levels of GII continued to be associated with higher odds of caregiver physical abuse (OR = 1.042; *p* < 0.001). More substantively, a 1% increase in GII was associated with a 4.2% increase in the odds of child physical abuse. GDP was not associated with physical abuse. MICS Round 5 was associated with lower odds of physical abuse (OR = 0.935; *p* < 0.05). Higher levels of household wealth were associated with lower odds of physical abuse. Urban residence was associated with higher odds of physical abuse (OR = 1.116; *p* < 0.001). A higher number of household members was associated with higher odds of physical abuse (OR = 1.034; *p* < 0.001). Education was to some degree associated with physical abuse, in that having a secondary or higher education was associated with lower odds of physical abuse (OR = 0.867; *p* < 0.001). When the survey respondent was male, this was associated with lower odds of physical abuse (OR = 0.864; *p* < 0.001). Similarly, when the survey respondent was someone other than the biological parent, this was associated with lower odds of physical abuse. The respondent’s belief that physical punishment is necessary was associated with higher odds of physical abuse (OR = 2.717; *p* < 0.001). Female children were less likely to be abused than male children (OR = 0.838; *p* < 0.001). Model 4 was the full model with the GII and child gender interaction. The statistically significant, positive interaction between GII and being a female child suggested that the association of GII and physical abuse was stronger for girls than boys (OR = 1.007; *p* < 0.001).

**Figure 2:**
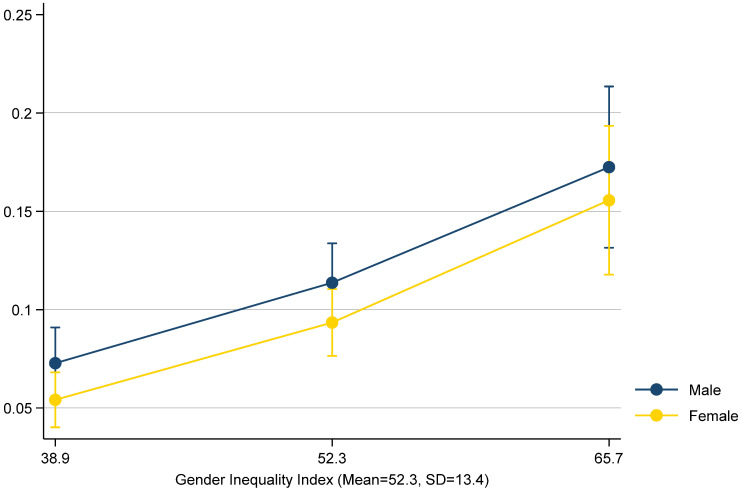
Predicted Probabilities of Caregiver Physical Abuse by Levels of GII.

In order to more fully explore differences in outcomes for male and female children, we examined predicted probabilities of physical abuse for male and female children at different levels of gender inequality (Figure 2). We were also aware that in nonlinear models, such as logistic regression models, interaction terms may not accurately reflect the presence of an interaction in the underlying data [48,49]. We therefore calculated probabilities of physical abuse for each child gender at the mean level of GII as well as 1 standard deviation above the mean and 1 standard deviation below the mean. Tests of these predicted probabilities indicated that the probability of physical abuse increased as GII increased. The predicted probabilities of physical abuse were not statistically different by child gender at the mean of GII, 1 SD below the mean, and 1 SD above the mean. However, when we compared the difference in physical abuse by child gender at the mean level of GII with the difference in abuse by gender at 1 SD above the mean of GII, there was a slightly smaller difference between the genders at the higher level of GII (*p* = 0.06). 

**Figure 3:**
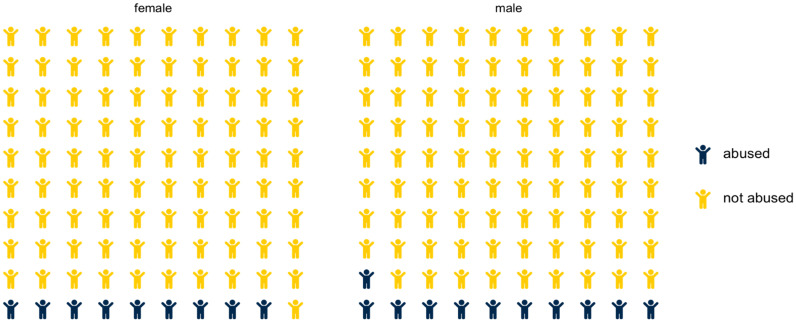
Physical Abuse Among Hypothetical Samples of 100 Children at Mean Levels of Gender Inequality. Note: Results suggest that at mean levels of GII, 11 male children and 9 female children would be subject to abuse. Were a similar figure to be constructed for countries where GII was 1 standard deviation above the mean, 17 male children and 16 female children would be subject to abuse. At 1 standard deviation below the mean, 7 male children and 5 female children would be subject to abuse.

Presentation of predicted probabilities may be enhanced by use of natural frequencies [50,51]. Therefore, we created waffle plots to show how the calculated predicted probabilities at mean levels of GII might apply to hypothetical samples of 100 children [52]. Figure 3 suggests that at the mean level of GII, 11 male children and 9 female children would be subject to physical abuse. Were a similar figure to be constructed for countries where GII was 1 standard deviation above the mean, 17 male children and 16 female children would be subject to physical abuse. At 1 standard deviation below the mean, 7 male children and 5 female children would be subject to physical abuse.

## 4. Discussion

Reducing gender disparities and children’s exposure to parental violence are global health priorities and notable Sustainable Development Goals [53]. We used nationally representative reports of caregiver behaviors in the UNICEF MICS data to assess the association between country-level GII and children’s exposure to physical abuse at the household level. In support of prior literature and study hypotheses, our findings based on 51 LMICs provide evidence that higher levels of gender inequality were associated with increased levels of caregiver reported physical abuse against children. Results from moderation analyses provide evidence of a statistical interaction between gender inequality and child gender such that the association of gender inequality with physical abuse is stronger for female children than for male children. 

Notably, our analysis was limited to the most severe forms of parental physical violence against children. The CTS operationalizes beating children up as “very severe assault, severe physical maltreatment” and slapping children’s face, head, or ears as “severe assault, physical maltreatment” [45]. The CTS defines hitting children’s bottom with an object as physical punishment, not physical maltreatment. Prior research on physical abuse in LMICs using data from the MICS and the Demographic and Health Surveys [36,39] used a broader definition and included hitting children with an object in the measure of physical abuse. In contrast, our analyses excluded hitting children with an object because MICS questionnaires did not differentiate where on the body the child was hit. Thus, our results, which focused on the associations between gender inequality and the most severe forms of physically abusive parenting behavior in LMICs, could be interpreted as conservative estimates as nearly 30% of children in LMICs were hit with an object on the bottom or elsewhere [24]. 

### 4.1. Association between Gender Inequality and Physical Abuse

Societies with high rates of gender inequity report a lack of protection for women and children from violent family interactions [29]. Our results are consistent with previous literature demonstrating the bivariate associations between gender inequity and children’s exposure to physical abuse in LMICs [5,36]. Our findings expand this evidence based on the associations of macro contexts with physically aggressive parenting behaviors by using a developmental ecological and multilevel approach that accounts for family-, parent- and child-level factors in analyses. In particular, gender inequality was positively associated with children’s exposure to physical abuse over and above favorable attitudes toward physical punishment, a strong and consistent predictor of caregivers’ use of physically aggressive behaviors in LMICs [41,54,55]. These results highlight the critical need for interventions to concurrently address structural gender disparities and caregiver perceptions regarding violence against children in order to reduce rates of caregiver physical abuse in LMICs. 

In this analysis, structural or macro level factors indicative of discriminatory access to educational and economic opportunities for women had a complex relationship with rates of child physical abuse. As noted, higher levels of gender inequality were associated with both higher odds and higher probabilities of physical abuse. Interestingly, GDP was not associated with rates of abuse. However, this is not to say that economic factors were not associated with rates of abuse as household wealth—a relative within-country measure—was associated with physical abuse. Similarly, higher levels of education were associated with lower rates of physical abuse. 

### 4.2. Association of Gender Inequality and Physical Abuse by Child Gender

Our finding that male and female children are equally at risk for physical abuse is consonant with some prior research [43]. Notably, for both boys and girls, rates of physical abuse increase as gender inequality increases, or conversely might be said to decrease as gender equality decreases. These findings further illustrate the complexity of interaction terms within nonlinear models, and the necessity of employing advanced quantitative analysis to explore the results of such models in order to avoid making incorrect conclusions about important outcomes, such as physical abuse. As noted, the main effect and interaction terms in the logistic regression are both statistically significant, and the magnitude of the two coefficients suggests that female children are at lower risk of abuse but that the risk of abuse becomes more salient for female children as gender inequality increases. A more naive reading of the regression estimates might suggest that female children are less at risk of physical abuse than male children. However, as prior research has pointed out [48,49], it is not always acknowledged but mathematically incorrect to assume that the regression coefficient for an interaction term in a nonlinear model provides direct information about the underlying processes. Instead, as suggested by Long and Freese [56] we calculated predicted probabilities of physical abuse for male and female children at different levels of gender inequality. Tests of these predicted probabilities against each other suggested that—in contrast to parameter estimates from the regression equation—rates of abuse for male and female children are statistically indistinguishable, at all levels of gender inequality, although there is some slight evidence that rates of abuse converge as gender inequality increases. 

### 4.3. Implications for Policy and Practice

The damaging consequences of physical violence have no borders. Yet, research continues to suggest that violence against children may be more pervasive in low-resource countries than in economically advanced countries [57,58], which exacerbates global inequalities between populations and perpetuates long-lasting social and economic disparities. Our findings suggest that improving women’s health, political, and economic outcomes as stated in the United Nations Sustainable Development Goal (SDG) 5, “Achieve gender equality and empower all women and girls” would protect children from physical abuse as urged by the SDG 16 Target 2, “End abuse, exploitation, trafficking and all forms of violence against and torture of children” [59,60]. Put differently, eliminating gender discriminatory legislation and practices would empower women economically and politically, thereby strengthening their caregiving roles and promote nurturing, non-physical child rearing practices. Furthermore, advancing gender equality would reduce gender-based violence and exploitation in the family context that are grounded in discriminatory social norms. The empowerment of women would potentially prevent the spillover of violence against children. 

### 4.4. Limitations and Considerations for Future Research

Despite the strengths of our study, particularly the use of a large global data that includes country- and household-level information pertaining to parenting and child development, our findings should be interpreted in light of the following limitations. First, our results are subject to social desirability and reporting biases as our measures of physically abusive caregiver behaviors relied on the household respondent’s self-report. This may have resulted in an underreporting of physical abuse, which may be a more prevalent form of family violence that children are exposed to than reported in this study. Yet arguably, this limitation is partly addressed by the way in which the CTS measured parenting behaviors—namely, by solely asking to report the occurrence of 11 types of parenting behaviors including psychological and physical aggression and non-aggressive discipline, rather than focusing on the possible negative effects of parenting behaviors on the child [39]. Another limitation is that the binary definition of female and male children within MICS fails to consider children with diverse gender identities and the ways in which gender stereotypes and roles that stigmatize non-binary or transgender identities may affect physically abusive parenting behaviors. Finally, our analyses were cross-sectional and correlational; we cannot speak to causality and there might be some factors that would confound the association between the GII and physical abuse that were not measured in our study. Future research should examine the associations between the specific dimensions of the GII and physical abuse and the mechanisms that might explain how gender inequality might be associated with physically abusive behaviors. 

## 5. Conclusions

Gender inequality is an important predictor of higher rates of child physical abuse. Rates of physical abuse appear to be equivalent across child gender, and to increase as gender inequality increases. Consistent with former Secretary-General of the United Nations (UN) Kofi Annan’s words, “When women thrive, all of society benefits, and succeeding generations are given a better start in life,” [61] our findings indicate that gender equality benefits all children, especially in low-resource settings where child care responsibilities primarily fall on women. Interventions to reduce child physical abuse would likely benefit from exploring ways to intervene at the macro level, especially with factors such as gender inequality, including stronger societal investments to increase women’s economic and educational opportunities and reducing discriminatory gender norms. 

## Figures and Tables

**Table 1:** Descriptive Characteristics of Study Participants (*N* = 424,414).

Variables	Mean or %	SD	Min	Max
Caregiver Physical Abuse				
No	92.3			
Yes	7.7			
Gender Inequality Index	52.3	13.4	14.9	74.5
GDP (1000 US$)	3.48	3.20	0.33	19.03
Physical Punishment is Necessary				
No	79.8			
Yes	16.6			
Don’t know/No opinion	3.6			
Respondent is male	73.2			
Respondent relationship with child				
Biological parent	72.0			
Grandparent	19.4			
Other	8.6			
Respondent education				
None	14.0			
Primary	31.2			
Secondary or higher	54.8			
Number of household members	5.2	2.4	1	50
Wealth Index				
Poorest	20.3			
Second	21.6			
Middle	19.9			
Second Richest	19.9			
Richest	18.3			
Urban residence	56.0			
MICS Round				
Round 4	45.3			
Round 5	54.7			
Child age (years)	7.5	4.0	1	17
Child is male	52.2			

Note: Household- and individual-level variables are weighted.

**Table 2:** Multilevel Logistic Regression Models Predicting Physical Abuse.

Predictors	Model 1	Model 2	Model 3	Model 4
	Country	Household	Individual	Child Sex Moderation
Gender Inequality Index	1.051 ***	1.048 ***	1.042 ***	1.038 ***
	(0.008)	(0.008)	(0.008)	(0.008)
GDP (1000 USD)	0.971	0.966	0.969	0.969
	(0.028)	(0.028)	(0.027)	(0.027)
MICS Round 5	0.969	0.980	0.935 *	0.935 *
	(0.028)	(0.028)	(0.027)	(0.027)
Wealth Quintile				
Second Poorest		0.847 ***	0.893 ***	0.893 ***
		(0.011)	(0.012)	(0.012)
Middle		0.780 ***	0.850 ***	0.850 ***
		(0.011)	(0.012)	(0.012)
Second Richest		0.703 ***	0.792 ***	0.793 ***
		(0.011)	(0.013)	(0.013)
Richest		0.586 ***	0.713 ***	0.714 ***
		(0.010)	(0.013)	(0.013)
Urban Residence		1.108 ***	1.116 ***	1.116 ***
		(0.013)	(0.014)	(0.014)
Household Members		1.031 ***	1.034 ***	1.034 ***
		(0.002)	(0.002)	(0.002)
Respondent Education (=None)				
Primary			1.001	1.001
			(0.012)	(0.012)
Secondary +			0.867 ***	0.867 ***
			(0.013)	(0.013)
Respondent is Male			0.864 ***	0.864 ***
			(0.012)	(0.012)
Respondent is Biological Parent				
Grandparent			0.770 ***	0.769 ***
			(0.011)	(0.011)
Other			0.854 ***	0.852 ***
			(0.016)	(0.016)
Physical Punishment is Necessary (=No)				
Yes			2.717 ***	2.716 ***
			(0.027)	(0.027)
Don’t know/No opinion			1.658 ***	1.658 ***
			(0.046)	(0.046)
Child Age (Years)			1.001	1.001
			(0.001)	(0.001)
Child is Female			0.838 ***	0.547 ***
			(0.008)	(0.030)
GII × Female Child				1.007 ***
				(0.001)
Constant	0.011 ***	0.012 ***	0.018 ***	0.021 ***
	(0.005)	(0.006)	(0.008)	(0.010)
Observations	424,414	424,414	424,414	424,414
Number of countries	51	51	51	51

Note: Standard errors in parentheses; * *p* < 0.05, *** *p* < 0.001.

## Data Availability

Not applicable.
